# Draft Genome Sequence of Xanthomonas sacchari Strain LMG 476

**DOI:** 10.1128/genomeA.00146-15

**Published:** 2015-03-19

**Authors:** Isabelle Pieretti, Stéphanie Bolot, Sébastien Carrère, Valérie Barbe, Stéphane Cociancich, Philippe Rott, Monique Royer

**Affiliations:** a CIRAD, UMR BGPI, Montpellier, France; b INRA, Laboratoire des Interactions Plantes Micro-organismes (LIPM), UMR 441, Castanet-Tolosan, France; c CNRS, LIPM, UMR 2594, Castanet-Tolosan, France; d CEA, DSV/IG/Genoscope, Centre National de Séquençage, Evry, France; e University of Florida, IFAS, Everglades Research and Education Center, Belle Glade, Florida, USA

## Abstract

We report the high-quality draft genome sequence of Xanthomonas sacchari strain LMG 476, isolated from sugarcane. The genome comparison of this strain with a previously sequenced X. sacchari strain isolated from a distinct environmental source should provide further insights into the adaptation of this species to different habitats and its evolution.

## GENOME ANNOUNCEMENT

To date, the Xanthomonas genus accounts for 37 plant-associated species and subspecies according to the current prokaryotic nomenclature listing at the Leibniz Institute DSMZ (http://www.dsmz.de/bacterial-diversity/prokaryotic-nomenclature-up-to-date.html). However, the recently proposed new species X. maliensis isolated from rice could be added to this list (1). The xanthomonads’ taxonomy evolves regularly according to the methodologies used for description, resulting in changes in the distribution and delimitation of species, as well as in the reallocation of pathovars within species. In 1995, on the basis of DNA-DNA hybridization analyses, 20 Xanthomonas species (genomospecies) were proposed, elevating many X. campestris pathovars to the rank of species or placing them in already described species (2). This work resulted also in the repositioning of strains LMG 471 and LMG 476, isolated from sugarcane in Guadeloupe in 1980. These two strains, initially considered to belong to the sugarcane pathogenic species X. albilineans, were renamed as a new Xanthomonas species called X. sacchari (2). Thereafter, phylogenetic trees based on 16S ribosomal DNA or 16S-23S ribosomal DNA intergenic sequences, as well as multilocus sequence analyses, supported the distinction of both LMG 471 and LMG 476 strains from the X. albilineans species, nevertheless confirming that the X. sacchari species is genetically close to X. albilineans (3–5). X. albilineans is a sugarcane pathogen causing leaf scald disease, whereas the pathogenicity of X. sacchari remains unknown. An additional X. sacchari strain, NCPPB4393, isolated from an insect collected on a banana plant in Tanzania in 2007, has recently been sequenced (6, 7). The lack of information regarding both the adaptation of X. sacchari to two different habitats (insect and plant) and the specific genomic traits of this species raised the interest to compare strain NCPPB4393 with an X. sacchari LMG strain. We report herein the draft genome sequence of X. sacchari strain LMG 476, which is considered the type strain of this species (2).

Strain LMG 476 was sequenced using a Solexa HiSeq (Illumina) sequencer (Genoscope, France). The shotgun sequencing yielded 72,261,076 76-bp paired-end reads with an insert size of 200 bp. A combination of Velvet (8), SOAPdenovo, and SOAPGapCloser (9) yielded 74 contigs larger than 500 bp and a largest scaffold of 4,597,345 bp (using X. albilineans strain GPE PC73 as a reference for guiding the final scaffolding step) for a total assembly size of 4,898,860 bp (excluding scaffolding gaps), corresponding to 1,121× coverage. The average G+C content is 69%.

Calculation of the average nucleotide identity (ANI) was performed between the genome sequences of strain LMG 476, strain NCPPB4393, and strain GPE PC73 of X. albilineans using the JSpecies calculator (http://www.imedea.uib.es/jspecies) (10) based on the BLASTn method (ANIb) and the MUMer algorithm (ANIm). The ANI values between strains LMG 476 and NCPPB4393 were above 99%, whereas those between strains LMG 476 or NCPPB4393 and GPE PC73 were below 87%, thus confirming that strain NCPPB4393 belongs to the X. sacchari species. The genome sequence of strain LMG 476 can now be used to elucidate the genomics and evolution of this species of Xanthomonas, whose habitats remain unclear, especially because strain NCPPB4393 was isolated from an insect.

### Nucleotide sequence accession numbers.

This whole-genome shotgun project has been deposited at DDBJ/EMBL/GenBank under the accession number JXQE00000000. The version described in this paper is version JXQE01000000.
